# Comparison Between Interlaminar and Transforaminal Endoscopic Lumbar Discectomy for Sciatic Scoliosis Secondary to Lumbar Disc Herniation: A Retrospective Study With a Minimum 1 Year Follow‐Up

**DOI:** 10.1111/os.70447

**Published:** 2026-10-16

**Authors:** Shicheng Zheng, Xincheng Cai, Bolu Zhang, Qinghua Zhao, Zongshan Hu, Bin Wang, Zezhang Zhu, Yong Qiu, Xu Sun

**Affiliations:** ^1^ Division of Spine Surgery, Department of Orthopedic Surgery Nanjing Drum Tower Hospital, Affiliated Hospital of Medical School, Nanjing University Nanjing China

**Keywords:** interlaminar, lumbar disc herniation, percutaneous endoscopy, sciatica, scoliosis, transforaminal

## Abstract

**Objective:**

This study aims to compare the clinical and radiographic outcomes of interlaminar and transforaminal endoscopic lumbar discectomy (IELD and TELD) in the treatment of lumbar disc herniation (LDH) with sciatic scoliosis.

**Methods:**

Patients with sciatic scoliosis secondary to LDH who had undergone IELD or TELD between January 2022 to June 2024 were reviewed. Radiographic parameters, including Cobb angle, apical vertebral translation (AVT), and coronal balance distance (CBD), were measured preoperatively and postoperatively to assess curve evolution. Clinical outcomes were evaluated using the Visual Analog Scale (VAS) and the Oswestry Disability Index (ODI). Meanwhile, perioperative data were also collected. Comparisons between the groups were made by independent *t*‐tests and *χ* ^2^ tests.

**Results:**

A total of 134 patients in the IELD group and 103 patients in the TELD group were included in this study. There was no significant difference in intraoperative blood loss (13.4 ± 7.8 mL vs. 11.5 ± 7.1 mL, *p* = 0.067) and postoperative length of stay (2.5 ± 0.6 days vs. 2.5 ± 0.8 days, *p* = 0.378) between the two groups; however, the IELD group demonstrated shorter operation time (87.2 ± 17.4 min vs. 96.1 ± 23.3 min, *p* = 0.001) and fewer intraoperative fluoroscopy times (4.9 ± 0.7 vs. 10.3 ± 2.7, *p* < 0.001) compared to the TELD group. Both groups showed significant improvements in VAS and ODI scores (*p* < 0.05), with no significant differences between the groups. Before surgery, 86.9% (206/237) patients had a disc herniation at the convex side of the lumbosacral curve and 86.5% (205/237) patients showed a trunk shift toward the opposite side of disc herniation. After surgery, 91.8% (123/134) of the IELD group and 90.3% (93/103) of the TELD group achieved resolution of scoliosis (AVT ≤ 10 mm) within 12 months. At the final follow‐up, the AVT, Cobb angle, CBD, and SVA in the IELD group were 2.7 ± 2.0 mm, 1.0° ± 0.8°, 1.2 ± 0.9 mm, and 3.4 ± 3.1 mm, respectively, with no significant differences compared to the 2.8 ± 2.7 mm, 1.1° ± 0.8°, 1.2 ± 0.8 mm, and 3.7 ± 3.5 mm in the TELD group.

**Conclusions:**

Both IELD and TELD are effective and safe for LDH with sciatic scoliosis, with comparable radiographic and clinical outcomes in terms of scoliosis correction. IELD is associated with shorter operation time and fewer intraoperative fluoroscopy times in this cohort.

## Graphical abstract

In this study, we aimed to evaluate and compare the clinical and radiographic outcomes of interlaminar endoscopic lumbar discectomy (IELD) and transforaminal endoscopic lumbar discectomy (TELD) in patients with lumbar disc herniation (LDH) with sciatic scoliosis, an area where evidence is limited and clinical decision‐making remains challenging.

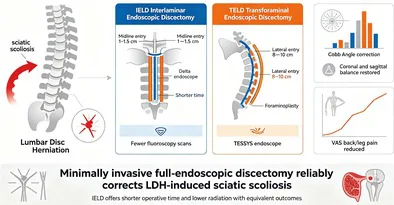

## 1 Introduction

Lumbar disc herniation (LDH) is a prevalent condition that affects 1%–5% of the population annually [1] and most commonly presents with low back pain, often accompanied by leg pain [2]. Besides, some patients may also experience sciatica. To alleviate this pain, they may exhibit sciatic scoliosis [3].

Sciatic scoliosis is a condition characterized by a lateral or forward trunk tilt, caused by nerve root irritation [4]. It is also known by several other terms, including scoliotic list [3], sciatic scoliotic list [3, 4, 5], lumbosacral list [6], and trunk list [7, 8, 9]. The incidence of sciatic scoliosis in patients with LDH has been reported to range from 1.4% to 32.0% in various studies [10, 11]. Patients with sciatic scoliosis are typically aged between 20 and 50 years, in generally good health, and often possess a muscular and vigorous constitution [12]. Although the exact mechanism is not fully understood, sciatic scoliosis has been considered not as a structural curve, but as a compensatory attempt of the body to alleviate nerve irritation. Once nerve irritation is timely eliminated, scoliosis can be corrected spontaneously [13].

Over the years, several methods have been explored for the management of sciatic scoliosis secondary to LDH. Gillan et al. [7] used the McKenzie method to manage patients with scoliotic scoliosis and found that it may help address the scoliotic scoliosis but is ineffective in improving clinical condition. According to the study by Zhu et al. [13], persistent scoliosis may be the initiating factor for structural scoliosis, and therefore, early surgical intervention should be considered. They treated 26 adolescents with sciatic scoliosis by open discectomy and found that all patients had significant improvement in scoliosis after surgery. Recently, percutaneous endoscopic lumbar discectomy (PELD) has attracted more and more attention as a minimally invasive spinal surgery [10, 11, 14], including two classic approaches, such as interlaminar endoscopic lumbar discectomy (IELD) and transforaminal endoscopic lumbar discectomy (TELD). Compared with traditional open surgical methods, PELD has the advantages of higher safety, less trauma and faster postoperative recovery [15]. Previous studies have shown that PELD is an effective method for the treatment of LDH with sciatic scoliosis [10, 11, 14], but no comparative studies on the approach between interlaminar and transforaminal have been reported.

Therefore, the purpose of this study was to evaluate the clinical and radiographic outcomes between IELD and TELD in the treatment of LDH with sciatic scoliosis.

## 2 Methods

### 2.1 Study Design and Patient Population

This study followed the STROBE (Strengthening the Reporting of Observational Studies in Epidemiology) guidelines. We retrospectively reviewed a cohort of LDH patients with sciatic scoliosis who underwent IELD or TELD at Nanjing Drum Tower Hospital from January 2022 to June 2024 (Figure 1). This study was approved by the ethics committee of our hospital (Ethics approval number: 2026‐0095‐01) and strictly adhered to the ethical principles of the Declaration of Helsinki. Patients included in this study had to meet the following criteria: (1) age younger than 60 years; (2) unilateral radiating pain or leg numbness, with no significant improvement after more than 6 weeks of conservative treatment; (3) single‐level LDH according to symptoms, clinical signs and magnetic resonance imaging (MRI) or computed tomography (CT); (4) negative result of Adam's forward bending test; (5) Cobb angle ≥ 10° or apical vertebral translation (AVT) ≥ 20 mm [10, 13, 16, 17] on standing whole spine radiographs; and (6) received either IELD or TELD. Exclusion criteria were: (1) lumbar spinal stenosis, lumbar spondylolisthesis, or lumbar instability; (2) calcified LDH; (3) previous lumbar surgery at lesion segment; (4) MRI, CT and X‐ray findings indicating structural scoliosis (e.g., adolescent idiopathic scoliosis, degenerative scoliosis, etc.); and (5) less than 12‐months follow‐up.

**FIGURE 1:**
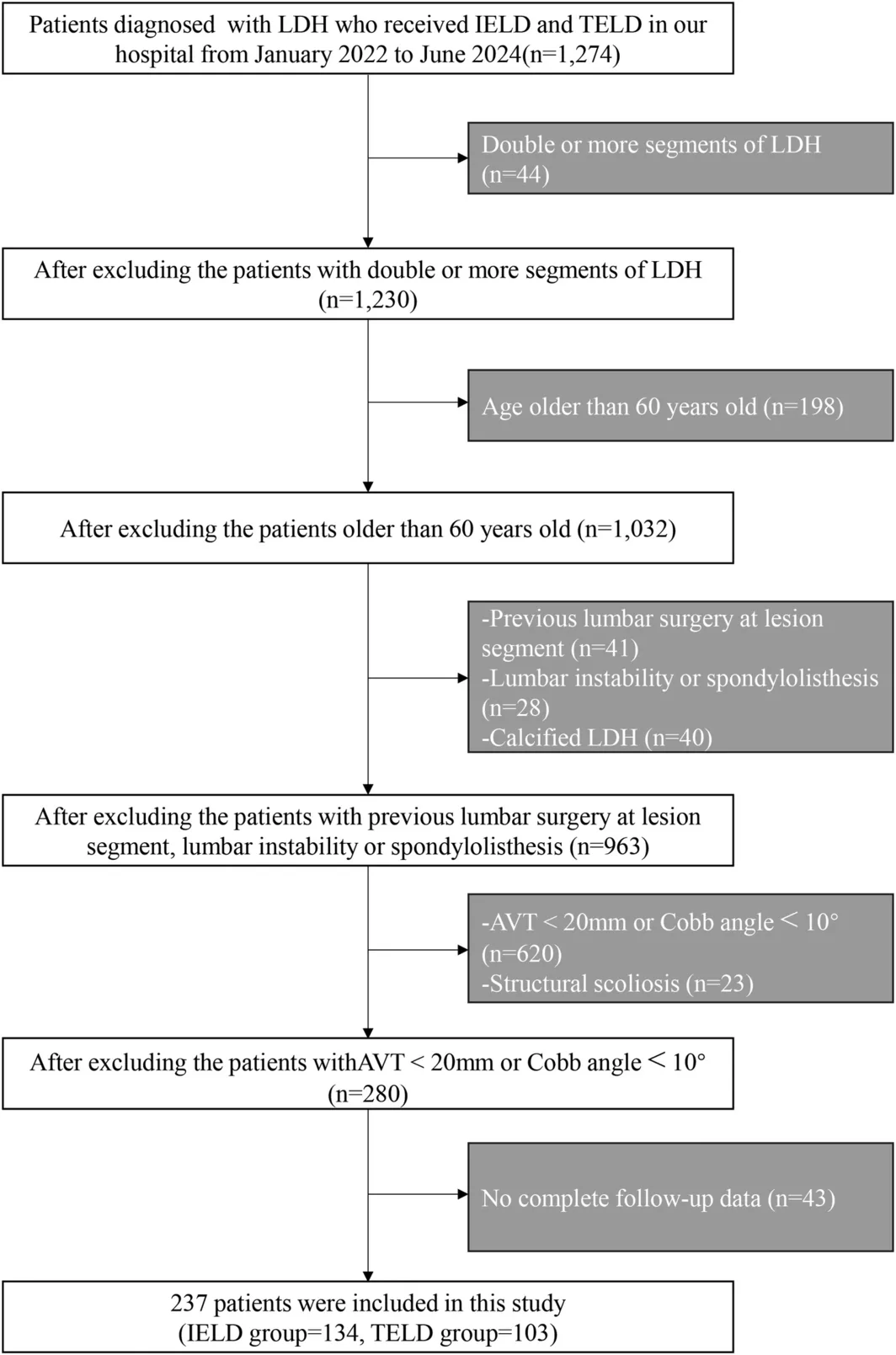
The study flow chart of patient selection process.

Patients were divided into IELD group and TELD group according to the surgery they received.

### 2.2 Radiographic Measurement

Preoperative and postoperative radiographs were obtained from all patients. Standing whole spine radiographs (Figure 2) were evaluated to assess the degree of scoliosis and spinal alignment. Radiological parameters were measured using Surgimap 2.0 software (Medicrea, New York).

**FIGURE 2:**
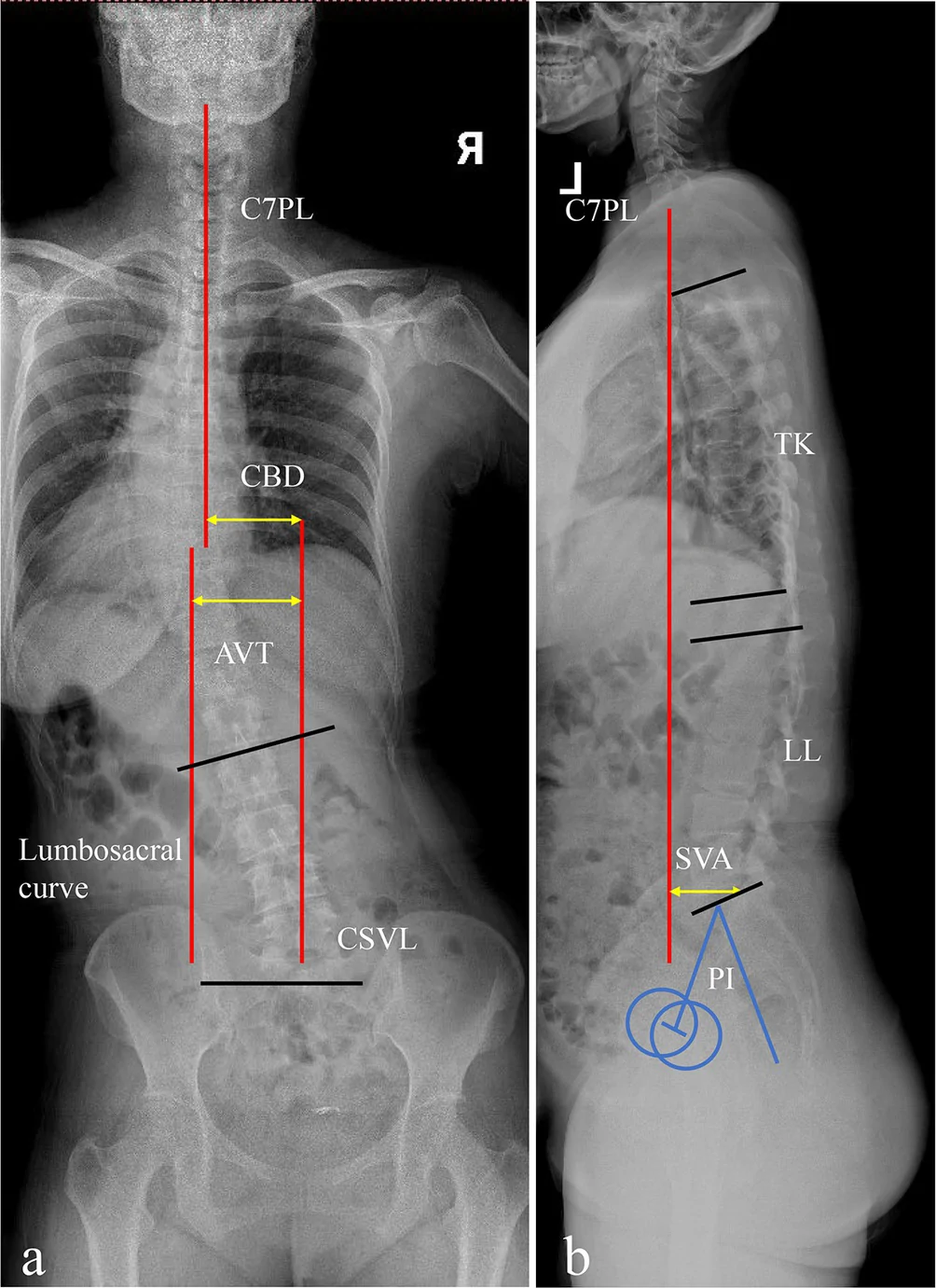
Schematic diagram of measured parameters. *Note:* All radiological measurements were made on standing whole spine radiographs.

On the coronal plane, the Cobb angle of lumbosacral curve was used to measure the degree of scoliosis. As for a lumbosacral curve that has its apex at L4, L5, or below, S1 was selected as the lower end vertebra for Cobb measurement. The apical vertebra (only thoracic and lumbar vertebrae) was identified as the most laterally deviated vertebra from the central sacral vertical line (CSVL). AVT was defined as the horizontal distance between the center of the apical vertebra and CSVL. Coronal balance distance (CBD) was calculated as the horizontal distance between the CSVL and the C7 plumb line (C7PL), with a shift to the left of the CSVL being considered a negative balance and a shift to the right considered a positive balance.

On the sagittal plane, pelvic incidence (PI), lumbar lordosis (LL), thoracic kyphosis (TK), and sagittal vertical axis (SVA) were measured. All radiological assessments were performed by two independent, experienced spine surgeons.

We used the resolution rate (RR) to evaluate the improvement of scoliosis at each follow‐up visit. Previous literature defined the recovery of scoliosis as when AVT ≤ 10 mm [17, 18]. RR is defined as the ratio of scoliosis to normal.

### 2.3 Surgical Technique

All surgeries were performed by the same spine surgeon (X.S.), who has over 1000 cases of microendoscopic discectomy experience, more than 2500 cases of PELD experience (including over 1200 cases of IELD and more than 1000 cases of TELD), and over 200 cases of unilateral biportal endoscopic discectomy experience.

Generally, patients with higher iliac crest, central or lateral recess stenosis, and lesion segment at L5/S1 received IELD general anesthesia. While patients with lesion segment at L4/5 received TELD under local anesthesia combined with intravenous sedation.

All procedures were placed in the prone position on a radiolucent table, with mild genuflexion and hip flexion. After appropriate sterilization and draping, C‐arm fluoroscopy was used for accurate localization of the surgical site. During the surgery, saline was used to continuously irrigate the surgical area, the radiofrequency electrode was used for hemostasis, and the ruptured annulus was ablated to promote its contraction and reshape it to reduce pressure on the nerve roots. The specific procedures are as follows.

#### 2.3.1 IELD

Generally, the insertion point should be targeted at the interlaminar space and located 1.0–1.5 cm lateral to the posterior middle line. But in cases with disc migration, the entry point was located at the midpoint of the line connecting the midpoint of the target segment and the furthest end of the migrated nucleus pulposus. After a 1.2‐cm‐length incision was made, a puncture needle was inserted into the interlaminar space. Then the soft tissue dilators and working cannula were placed along the guidewire, with accurate placement ensured by C‐arm fluoroscopy. Subsequently, the endoscope (iLESSYS‐Delta, Joimax, Germany) was introduced through the working cannula. The ligamentum flavum was identified, and laminotomy was performed by a high‐speed burr (Xishan, China) and a Kerrison rongeur under direct visualization. Then the dural sac, the transversing nerve root, and the disc were exposed. The herniated nucleus pulposus was then completely removed with forceps. The shoulder and axilla of the nerve root were thoroughly explored, ensuring that the compressed nerve root was set free.

#### 2.3.2 TELD

A puncture needle was inserted with the direction targeting at the tip of the superior articular process of the lesion segment. The entry point was usually located 8–10 cm lateral to the midline, and the puncture direction was slightly tilted (15°–25°) according to the patient's body size and the anatomical position of the herniated disc. Then a 7 mm‐length incision was made. The soft tissue dilator and the working cannula were gradually introduced. If necessary, visualization techniques were used to perform foraminoplasty, and part of the lateral‐ventral edge of the superior articular process was resected to enlarge the working channel, through which the endoscope (TESSYS, Joimax, Germany) was introduced. Under endoscopic guidance, the neural structures and herniated disc were identified. Subsequently, forceps were used to remove the nucleus pulposus, and the probe was used to ensure the relaxation of the nerve root. At the same time, attention was paid to patients' comfort.

For both surgical procedures, the completion criterion is defined as the observation of complete decompression of the nerve root and regular pulsation of the dural sac. Then the endoscope and the working cannula were removed, and the wound was carefully sutured with no need for drainage.

### 2.4 Postoperative Management

Standard postoperative treatment was administered, including medication measures to alleviate pain and reduce swelling. On the next day after surgery, all patients were guided to perform lumbar and lower limb functional exercises at the waist. After 24 h of bed rest, they were encouraged to sit and then to ambulate under the assistance of a waist brace. They were advised to avoid excessive lifting, bending, and twisting for 1 month, and also to incorporate moderate lumbar and back muscle exercises into their daily routine activities. All patients underwent regular standing radiographs of the total spine and MRI examinations at 3, 12 months postoperatively and the final follow‐up.

### 2.5 Clinical Outcome Assessment

Perioperative data, such as operation time, intraoperative blood loss, fluoroscopy times, and postoperative length of stay were recorded. Operation time was defined as the interval from skin incision to completion of wound closure.

Quality‐of‐life scores were assessed using the Visual Analog Scale (VAS) to evaluate pain relief and the Oswestry Disability Index (ODI) to evaluate functional disability. These outcomes were recorded before and after surgery. The modified Macnab criteria were evaluated to assess the satisfaction rate of patients at the final follow‐up.

### 2.6 Statistical Analysis

Data were analyzed using SPSS version 27.0 (SPSS, Chicago, IL, USA). All statistical data were used Shapiro–Wilk method to estimate the normal distribution of data. Continuous variables are expressed as means±standard deviations. Independent *t*‐test was used to compare the difference in age, BMI, follow‐up time, perioperative data, radiographic outcomes, VAS and ODI between IELD and TELD groups. Preoperative symptom duration showed a nonnormal distribution and was therefore expressed as median (interquartile range) and compared using the Mann–Whitney *U* test. Categorical variables were expressed as number. Gender, segment of LDH, side of LDH, comorbidities and modified MacNab criteria in two groups were evaluated by the *χ* ^2^ test. In addition, the Fisher's exact test was performed to analyze the relationship between side of disc herniation and the direction of lumbosacral curve and the trunk shift. Line and pie charts were generated using Prism 10 software (GraphPad Software, San Diego, CA, USA). Statistical significance was considered at a *p*‐value of < 0.05. Interobserver reliability for the Cobb angle, AVT, CBD, PI, SVA, TK, and LL was assessed using the intraclass correlation coefficient (ICC). ICC values of < 0.50, 0.50–0.75, 0.75–0.90, and > 0.90 were interpreted as indicating poor, moderate, good, and excellent reliability, respectively.

## 3 Results

### 3.1 Baseline Characteristics of the Patients

Finally, 237 patients were included in this study. There were 134 patients (79 males and 55 females) in the IELD group and 103 patients (65 males and 38 females) in the TELD group, respectively. The average age of the IELD group was 36.5 ± 10.8 years (15 adolescents, 79 young adults, 40 middle adults), while the average age of the TELD group was 37.1 ± 13.9 years (12 adolescents, 57 young adults, 34 middle adults). There were no significant differences between the two groups in terms of age, gender, BMI, segment of LDH, side of LDH, preoperative symptom duration, or comorbidities (*p* > 0.05, Table 1). The mean follow‐up time was 23.3 ± 6.5 months for the IELD group and 22.3 ± 6.1 months for the TELD group, with no significant difference (*p* = 0.276). The upper end vertebra (UEV) of the lumbosacral curve in both groups was mainly distributed at L2‐L3 (66.5% in the IELD group and 70.9% in the TELD group) with no significant difference (*p* = 0.689, Figure 3).

**TABLE 1:** Demographic and clinical characteristics of all the patients.

Variables	IELD group (*N* = 134)	TELD group (*N* = 103)	Statistic value	*p*
Age (years)	36.5 ± 10.8	37.1 ± 13.9	−0.34	0.736
Adolescent (≤ 18)	15 (11.2%)	12 (11.7%)		
Young adult (18–40)	79 (59.0%)	57 (55.3%)		
Middle adult (> 40)	40 (29.8%)	34 (33.0%)		
Gender (male/female)	79/55	65/38	0.42	0.516
BMI (kg/m^2^)	24.9 ± 4.3	25.0 ± 3.7	−0.33	0.744
Segment of LDH			0.58	0.340
L4/5	101 (75.4%)	83 (80.6%)		
L5/S1	33 (24.6%)	20 (19.4%)		
Side of LDH			1.11	0.292
Left	73 (54.5%)	49 (47.6%)		
Right	61 (45.5%)	54 (52.4%)		
Preoperative symptom duration (months)	3.0 (2.0–6.0)	3.0 (2.0–7.0)	−1.39	0.165
Comorbidities (*n*, %)				
Diabetes	3 (2.3%)	2 (1.9%)	0.00	1.000
Hypertension	10 (7.5%)	5 (4.9%)	0.43	0.414
Follow‐up time (months)	23.3 ± 6.5	22.3 ± 6.1	−1.25	0.276

Abbreviations: BMI, body mass index; LDH, lumbar disc herniation.

**FIGURE 3:**
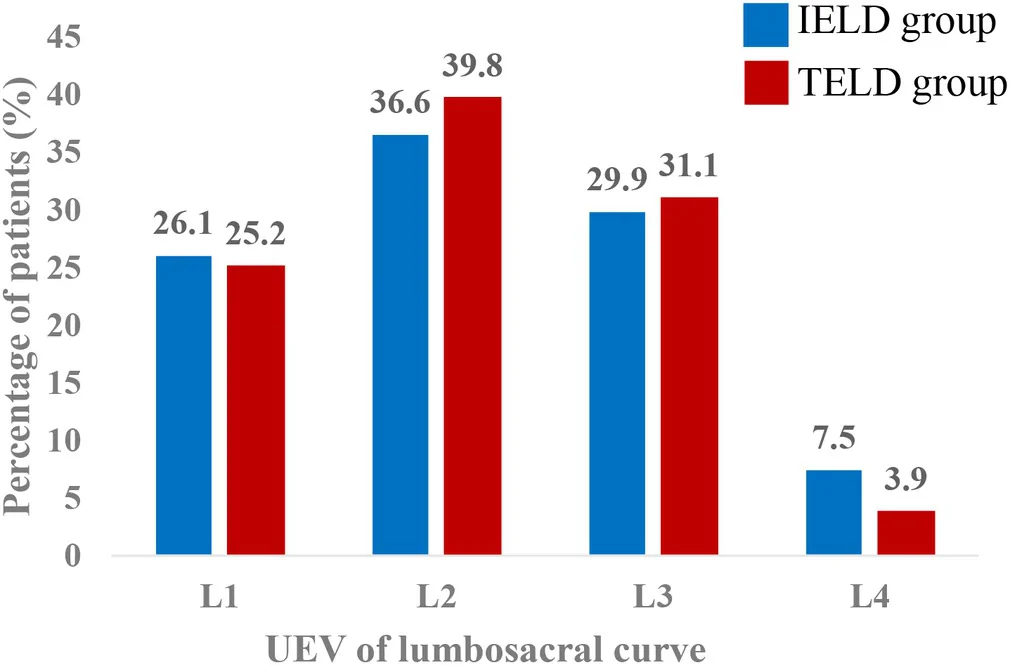
Distribution of the upper end vertebra in the lumbosacral curve among patients with sciatic scoliosis. *Note: χ* ^2^ tests, *p* = 0.689.

### 3.2 Association Between Side of Disc Herniation and Coronal Curve

As shown in Table 2, 86.9% (206/237) patients had a disc herniation at the convex side of the lumbosacral curve. There was a statistically significant association between the side of the lumbosacral curve and the side of the disc herniation according to Fisher's exact test (*p* < 0.001). Similarly, 86.5% (205/237) patients showed a trunk shift toward the opposite side of the disc herniation, and an apparent correlation between the direction of trunk shift and the side of disc herniation was noted (*p* < 0.001).

**TABLE 2:** Relationship between side of disc herniation and coronal curve.

	Side of disc herniation		*p*
	Left (*n* = 122)	Right (*n* = 115)	
Side of lumbosacral curve			
Left	19 (15.6%)	103 (89.6%)	< 0.001*
Right	103 (84.4%)	12 (10.4%)	
Direction of trunk shift			
Left	21 (17.2%)	104 (90.4%)	< 0.001*
Right	101 (82.8%)	11 (9.6%)	

* *p* < 0.05.

### 3.3 Perioperative Data

As shown in Table 3, significant differences were observed in operation time and fluoroscopy times between IELD and TELD groups. The IELD group had a shorter operation time compared to the TELD group (87.2 ± 17.4 min vs. 96.1 ± 23.3 min, *p* = 0.001). Additionally, the IELD group required significantly fewer intraoperative fluoroscopy times compared to the TELD group (4.9 ± 0.7 vs. 10.3 ± 2.7, *p* < 0.001). There was no significant difference in intraoperative blood loss (13.4 ± 7.8 mL vs. 11.5 ± 7.1 mL, *p* = 0.067) and postoperative length of stay (2.5 ± 0.6 days vs. 2.5 ± 0.8 days, *p* = 0.378) between the two groups.

**TABLE 3:** Comparison of perioperative data between two groups.

Variables	IELD group (*N* = 134)	TELD group (*N* = 103)	*t*	*p*
Operation time (min)	87.2 ± 17.4	96.1 ± 23.3	−3.26	0.001*
Intraoperative fluoroscopy times (*n*)	4.9 ± 0.7	10.3 ± 2.7	−19.48	< 0.001*
Intraoperative blood loss (mL)	13.4 ± 7.8	11.5 ± 7.1	1.93	0.067
Postoperative length of stay (days)	2.5 ± 0.6	2.5 ± 0.8	−0.88	0.378

* *p* < 0.05.

### 3.4 Radiographic Outcomes

All radiographic parameters demonstrated excellent interobserver reliability. The ICC values ranged from 0.943 to 0.971. Specifically, the ICCs were 0.962 for the Cobb angle, 0.971 for AVT, 0.954 for CBD, 0.943 for PI, 0.968 for SVA, 0.951 for TK, and 0.965 for LL. The corresponding 95% confidence intervals are presented in Table 4.

**TABLE 4:** Interobserver reliability of radiographic measurements.

Radiographic parameter	ICC	95% CI
Cobb angle	0.962	0.948–0.973
AVT	0.971	0.959–0.980
CBD	0.954	0.936–0.967
PI	0.943	0.921–0.959
SVA	0.968	0.955–0.977
TK	0.951	0.932–0.965
LL	0.965	0.951–0.975

*Note:* ICC, intraclass correlation coefficient.

Both the IELD and TELD groups showed significant improvements in coronal and sagittal parameters after surgery (*p* < 0.01, Table 5). In the IELD group, the Cobb angle reduced from 14.8° ± 3.5° preoperatively to 1.0° ± 0.8° at the final follow‐up. Similarly, the AVT decreased from 36.7 ± 21.3 mm to 2.7 ± 2.0 mm, and the CBD reduced from 29.2 ± 19.0 mm to 1.2 ± 0.9 mm. In the TELD group, the Cobb angle decreased from 14.7° ± 3.4° to 1.1° ± 0.8°, the AVT from 36.3 ± 26.4 mm to 2.8 ± 2.7 mm, and the CBD from 29.6 ± 19.5 mm to 1.2 ± 0.8 mm. No significant differences in the RR between the two groups were observed (*p* > 0.05, Figure 4).

**TABLE 5:** Comparison of evolution in coronal and sagittal parameters between two groups.

Variables	IELD group (*N* = 134)	TELD group (*N* = 103)	*t*	*p*
PI (°)	47.9 ± 8.5	49.5 ± 9.6	−1.37	0.171
Cobb angle (°)				
Preoperation	14.8 ± 3.5	14.7 ± 3.4	0.35	0.725
3‐Month follow‐up	4.2 ± 2.3	4.5 ± 2.5	−1.1	0.273
12‐Month follow‐up	1.2 ± 1.0	1.3 ± 1.0	−0.86	0.391
Final follow‐up	1.0 ± 0.8	1.1 ± 0.8	−1.61	0.110
AVT (mm)				
Preoperation	36.7 ± 21.3	36.3 ± 26.4	−0.5	0.616
3‐Month follow‐up	11.0 ± 7.0	12.2 ± 7.1	−1.25	0.214
12‐Month follow‐up	3.9 ± 3.0	4.5 ± 4.1	−1.45	0.148
Final follow‐up	2.7 ± 2.0	2.8 ± 2.7	−0.23	0.819
CBD (mm)				
Preoperation	29.2 ± 19.0	29.6 ± 19.5	−0.17	0.865
3‐Month follow‐up	8.5 ± 6.8	9.3 ± 7.4	−0.78	0.439
12‐Month follow‐up	2.4 ± 2.2	2.5 ± 1.6	−0.88	0.381
Final follow‐up	1.2 ± 0.9	1.2 ± 0.8	−1.29	0.199
SVA (mm)				
Preoperation	61.5 ± 54.7	69.5 ± 61.8	−1.06	0.337
3‐Month follow‐up	24.9 ± 17.9	26.9 ± 20.3	−0.65	0.519
12‐Month follow‐up	6.2 ± 5.2	7.6 ± 6.2	−1.04	0.297
Final follow‐up	3.4 ± 3.1	3.7 ± 3.5	−0.92	0.360
TK (°)				
Preoperation	20.9 ± 11.8	21.5 ± 11.5	−0.42	0.675
3‐Month follow‐up	25.9 ± 8.2	26.1 ± 8.9	−0.17	0.869
12‐Month follow‐up	30.9 ± 5.0	31.3 ± 5.6	−0.49	0.623
Final follow‐up	32.0 ± 4.5	32.3 ± 5.0	−0.43	0.666
LL (°)				
Preoperation	24.4 ± 14.7	24.3 ± 13.9	0.05	0.958
3‐Month follow‐up	33.3 ± 8.4	32.7 ± 9.1	0.54	0.591
12‐Month follow‐up	41.9 ± 4.8	42.5 ± 4.6	−0.91	0.361
Final follow‐up	42.0 ± 4.8	42.9 ± 4.2	−1.45	0.148

**FIGURE 4:**
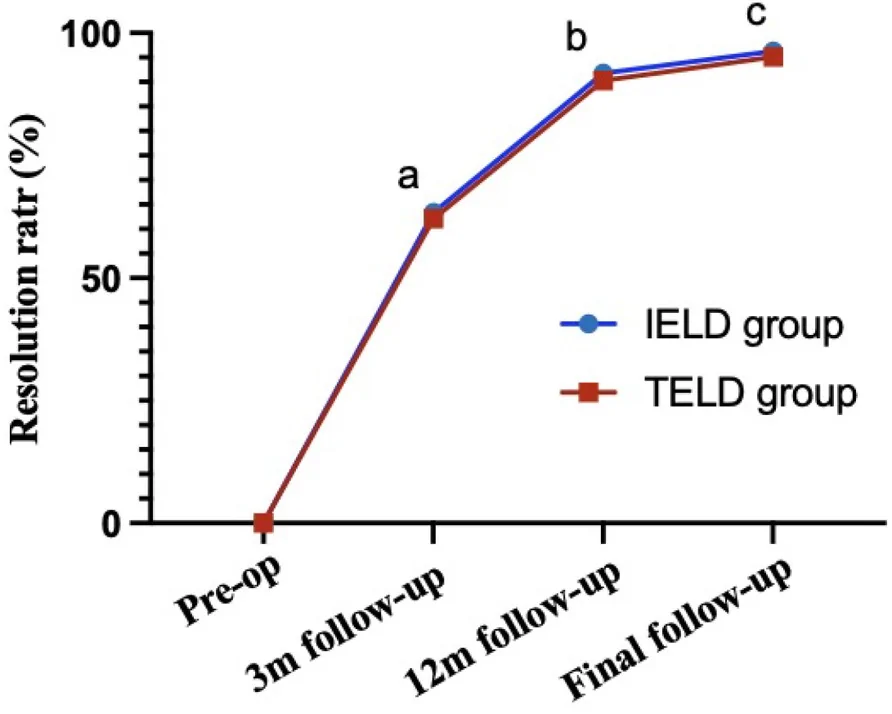
Line chart of the cumulative normalization rate of the patients with sciatic scoliosis during the follow‐up period. *Note:* (a) 63.4% vs. 62.1%, *p* = 0.838; (b) 91.8% vs. 90.3%, *p* = 0.687; and (c) 96.3% vs. 95.1%, *p* = 0.670.

Regarding sagittal alignment, no significant PI difference was found between the groups (47.9° ± 8.5° vs. 49.5° ± 9.6°, *p* = 0.109). The SVA decreased significantly in both groups: from 61.5 ± 54.7 mm to 3.4 ± 3.1 mm in the IELD group, and from 69.5 ± 61.8 mm to 3.7 ± 3.5 mm in the TELD group. Both groups showed significant increases in TK and LL, with the IELD group's TK rising from 20.9° ± 11.8° to 32.0° ± 4.5°, and LL increasing from 24.4° ± 14.7° to 42.0° ± 4.8°. Similarly, in the TELD group, TK increased from 21.5° ± 11.5° to 32.3° ± 5.0°, and LL from 24.3° ± 13.9° to 42.9° ± 4.2°.

Figure 4 shows a total of 91.8% (123/134) of the IELD group and 90.3% (93/103) of the TELD group achieved resolution of scoliosis within 12 months after operation. At the final follow‐up, the RR further increased to 96.3% (129/134) and 95.1% (98/103). Figures 5 and 6 show a typical case from the IELD group and the TELD group, respectively, and their sciatic scoliosis spontaneously corrected postoperatively.

**FIGURE 5:**
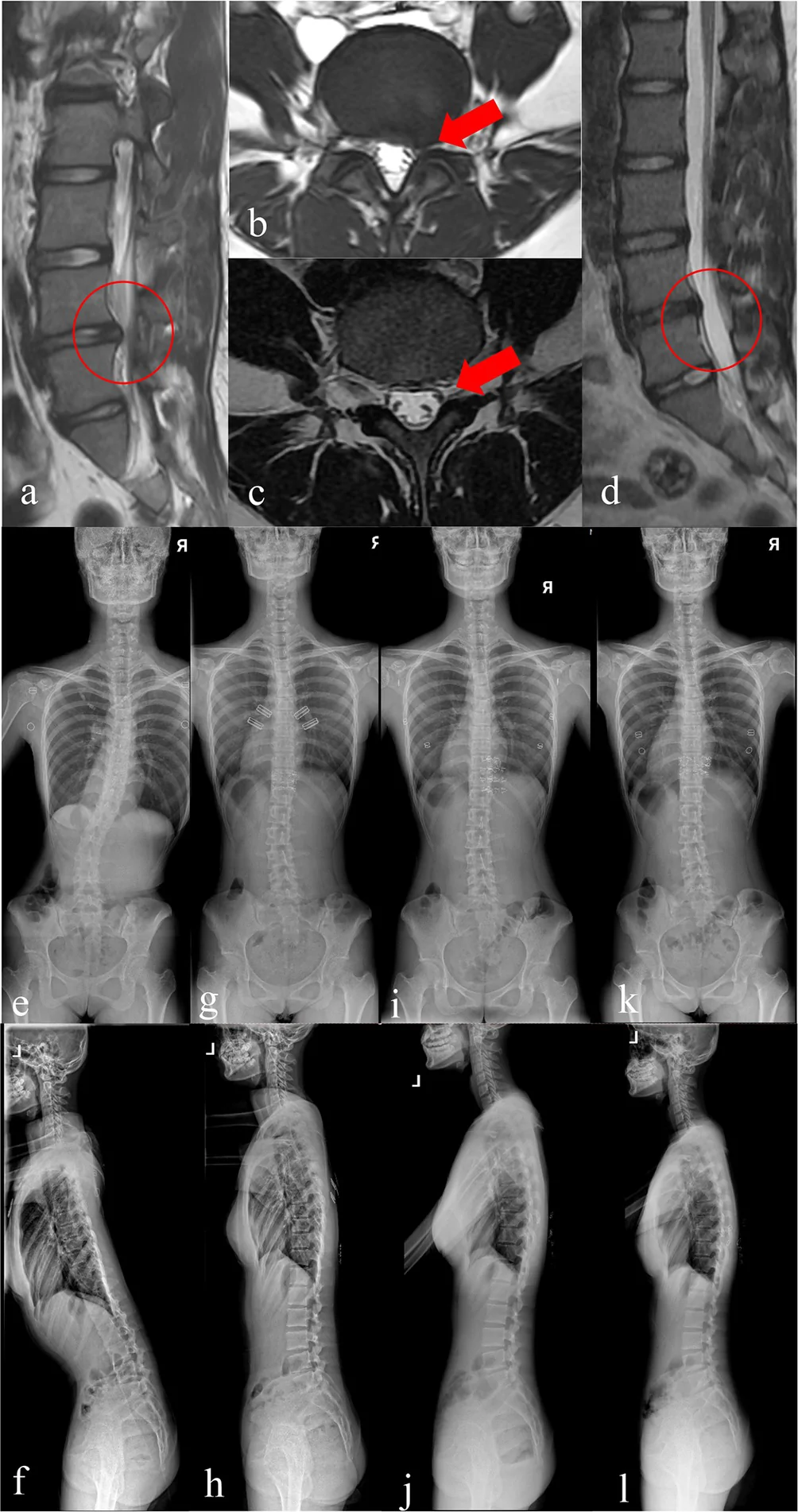
Evolution of a man aged 26 years with L5/S1‐disc herniation and sciatic scoliosis who underwent IELD. (a and b) Preoperative lumbar MRI shows L5/S1 disc herniation, compressing the left nerve root, as indicated by the red circle and arrow. (c and d) Postoperative lumbar MRI at 3 months shows complete removal of the disc, with no nerve root compression, as indicated by the red circle and arrow. (e and f) Preoperative standing whole spine radiographs show trunk shifting to the right, with a Cobb angle of 11° and AVT of 41.92 mm. There is also trunk forward with a very small TK and LL. (g and h) Postoperative standing whole spine radiographs at 3 months show that the scoliosis has nearly returned to normal, with the sagittal curvature also restored to physiological alignment. (i and j) Postoperative standing radiographs of the total spine at 12 months show that the scoliosis remains normal; and at the final follow‐up (k and l) at 18 months postoperatively, the spinal morphology is still maintained as normal.

**FIGURE 6:**
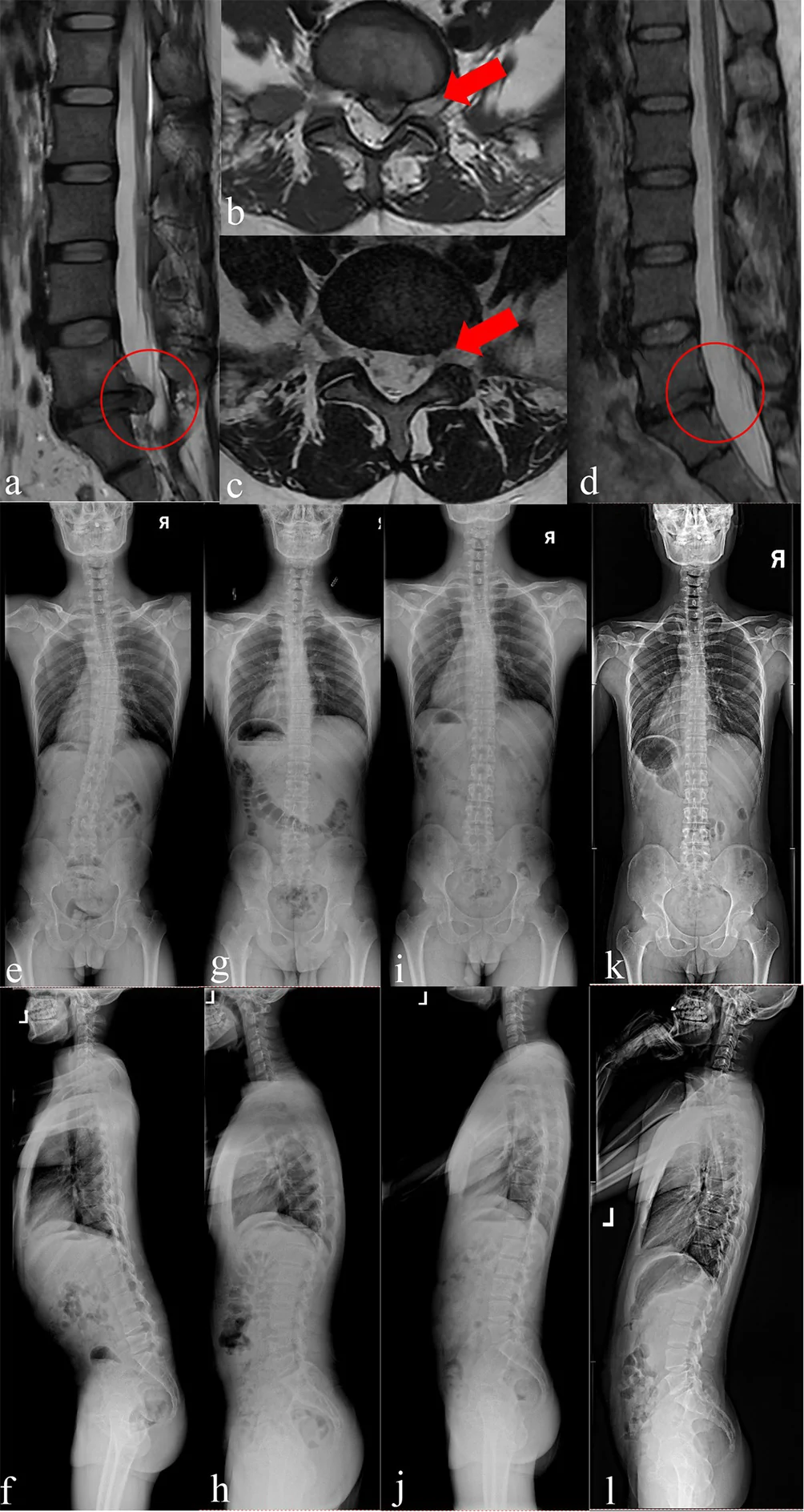
Evolution of a girl aged 15 years with L4/5‐disc herniation and sciatic scoliosis who underwent TELD. (a and b) Preoperative lumbar MRI shows L4/5 disc herniation, compressing the left nerve root, as indicated by the red circle and arrow. (c and d) Postoperative lumbar MRI at 3 months shows complete removal of the disc, with no nerve root compression, as indicated by the red circle and arrow. (e and f) Preoperative standing whole spine radiographs show trunk shifting to the right, with a Cobb angle of 18° and AVT of 55.89 mm. There is also trunk forward with a very small TK and LL. (g and h) Postoperative standing radiographs of the total spine at 3 months show that the scoliosis has nearly returned to normal, while the sagittal plane remains relatively straight. (i and j) Postoperative standing whole spine radiographs at 12 months show that the sagittal curvature has largely restored to physiological alignment; and at the final follow‐up (k and l) at 15 months postoperatively, the spinal morphology is still maintained as normal.

### 3.5 Clinical Outcomes

Table 6 shows that each group had significant improvement of ODI, VAS for back and leg pain at the final follow‐up (*p* < 0.001). However, there were no significant differences between the groups at any time point (*p* > 0.05).

**TABLE 6:** Comparison of patient‐reported outcomes between two groups.

Variables	IELD group (*N* = 134)	TELD group (*N* = 103)	*t* or *χ* ^2^ value	*p*
VAS for back pain				
Preoperation	6.4 ± 0.8	6.6 ± 0.9	−1.59	0.114
3‐Month follow‐up	2.4 ± 1.5	2.3 ± 1.5	0.95	0.344
6‐Month follow‐up	1.4 ± 1.1	1.4 ± 1.2	−0.37	0.714
12‐Month follow‐up	1.1 ± 1.3	1.1 ± 1.4	−0.17	0.864
Final follow‐up	1.1 ± 1.1	0.9 ± 1.0	0.93	0.353
VAS for leg pain				
Preoperation	7.1 ± 2.0	7.3 ± 1.8	−0.77	0.441
3‐Month follow‐up	1.9 ± 1.1	2.0 ± 1.4	−0.62	0.983
6‐Month follow‐up	1.6 ± 0.9	1.5 ± 0.9	0.83	0.409
12‐Month follow‐up	1.2 ± 1.3	1.1 ± 1.4	0.36	0.723
Final follow‐up	1.1 ± 1.2	1.1 ± 1.4	−0.09	0.931
ODI (%)				
Preoperative	52.3 ± 4.7	52.6 ± 5.1	−0.40	0.686
3‐Month follow‐up	15.9 ± 3.0	16.0 ± 3.0	−0.10	0.923
6‐Month follow‐up	9.5 ± 3.0	9.3 ± 2.6	0.56	0.578
12‐Month follow‐up	8.0 ± 1.1	8.1 ± 1.2	−0.62	0.534
Final follow‐up	7.0 ± 1.4	6.8 ± 1.4	0.89	0.376
Modified MacNab evaluation, *n* (%)			1.84	0.398
Excellent	63 (47.0%)	57 (55.3%)		
Good	61 (45.5%)	38 (36.9%)		
Fair	10 (7.5%)	8 (7.8%)		
Poor	0 (0.0%)	0 (0.0%)		
Excellent/good rate (%)	92.5%	92.2%		

As shown in Table 7, four patients in the IELD group and five patients in the TELD group experienced postoperative lower limb numbness, which recovered after 2 weeks of conservative treatment. Three patients in the IELD group and five patients in the TELD group developed lower back pain postoperatively, which alleviated after 3 weeks of bed rest. In the IELD group, four patients experienced recurrence, with two patients again demonstrating sciatic scoliosis, although to a lesser degree than the first episode. They received revision surgery, including TELD, micro endoscopic discectomy, mini‐open fenestration discectomy, and transforaminal lumbar interbody fusion, respectively. In the TELD group, two patients experienced recurrence, with one patient similarly demonstrating a less severe sciatic scoliosis compared to the first episode. They were treated with TELD and IELD, respectively. All patients showed significant symptom relief after receiving reoperations.

**TABLE 7:** Comparison of complications between two groups.

Variables	IELD group (*N* = 134)	TELD group (*n* = 103)	*χ* ^2^ value	*p*
Lower limb numbness, *n* (%)	4 (2.99)	5 (4.85)	0.16	0.687
Back pain, *n* (%)	3 (2.24)	5 (4.85)	0.55	0.458
Recurrence, *n* (%)	4 (2.99)	2 (1.94)	0.01	0.928

At the final follow‐up, both groups achieved a high rate of satisfaction assessed by the modified MacNab criteria, with 92.5% in the IELD group and 92.2% in the TELD group (*p* = 0.398).

### 3.6 Subgroup Analysis

Based on the segmental level, we further conducted subgroup analysis, including the L4/5 subgroup and the L5/S1 subgroup (Tables S1–S6). No results different from the overall analysis were observed in both groups. The radiographic outcomes, as assessed by PI, Cobb angle, AVT, CBD, SVA, TK, and LL, demonstrated a noticeable improvement in both groups, with no statistically significant difference. The clinical outcomes, as evaluated by VAS, ODI, and the modified MacNab criteria, demonstrated favorable results in both groups, with no statistically significant difference.

## 4 Discussion

This study was the first to compare the clinical and radiographical outcomes of IELD versus TELD in treating single‐level LDH with sciatic scoliosis. Our results revealed that more than 90% of patients in both groups achieved curve normalization within 12 months, and the final normalization rates were 96.3% in the IELD group and 95.1% in the TELD group, without a significant between‐group difference. Therefore, in patients with LDH‐related nonstructural sciatic scoliosis, the key therapeutic objective is adequate decompression of the symptomatic disc and nerve root rather than direct scoliosis correction.

In the coronal plane, Wu et al. [19] measured 68 patients with single‐level LDH accompanied by sciatic scoliosis, with an average Cobb angle of 16.5° and CBD of 26 mm. Yang et al. [18] analyzed 53 patients with single‐level LDH accompanied by sciatic scoliosis, with an average age of 35.9 years, an average Cobb angle of 15.3°, and a CBD of 19.0 mm. These findings are similar to those of our study. In the sagittal plane, most authors found an increase in SVA and a decrease in LL and TK [10, 17]. Some patients even lost their normal S‐shaped sagittal curve and showed the “straight line sign”. We also found that disc herniation at the L4/5 segment was more in patients with sciatic scoliosis (184/237, 77.6%), which was consistent with the research findings of Kim et al. [10]. The possible reason for this phenomenon is a relatively wider spinal canal at L5/S1 and the lack of a movable segment below L5/S1.

Zhu et al. [13] and Zhang et al. [17] have described the typical curve pattern of LDH with sciatic scoliosis. In adolescents, their curve pattern seems to develop a thoracic compensatory curve to maintain spinal coronal balance. As a result, they often present with long‐span curves (similar to King type IV in idiopathic scoliosis), making it crucial to distinguish them in order to avoid misdiagnosis. While in adults, they mostly exhibit a short lumbosacral curve accompanied by a long thoracic or thoracolumbar curve toward the opposite side, together with a relatively straight sagittal profile. According to Zhang et al. [17], this is due to the differences in spinal flexibility. We recommend that when encountering such cases in the outpatient setting, physicians should actively perform the Adam's forward bend test and straight leg raise test. Generally, patients with sciatic scoliosis will show curve disappearance after forward bending, which is distinctly different from patients with structural scoliosis [5]. However, the straight leg raise test will show a strong positive result.

LDH is considered a potential cause of sciatic scoliosis, which is a nonstructural scoliosis secondary to nerve root irritation [18]. Kim et al. [10] reported an incidence of sciatic scoliosis was approximately 18% in 164 adult patients. Tu et al. [14] found a higher incidence of sciatic scoliosis in adolescents, around 37.8%. Zhang et al. [17] reported that the incidence of sciatic scoliosis in adolescents and adults was approximately 34.8% (56/161) and 13.8% (128/926), respectively. In our study, the incidence of sciatic scoliosis was 18.6% (237/1274). The actual incidence should be higher because not all patients underwent standing radiographs of the total spine. However, the mechanical relationship between sciatic scoliosis and LDH is still unclear. Finneson et al. first proposed the “nerve root tension” hypothesis based on theoretical analysis, suggesting that when the disc herniation is located lateral to the nerve root, the spine bends toward the healthy side, whereas when the disc herniation is medial to the nerve root, the spine bends toward the affected side [4]. However, subsequent studies [3, 6] have reported clinical outcomes that contradicted Finneson's hypothesis.

Previous studies reported that in patients with sciatic scoliosis, 66.7%–84.6% of disc herniations occurred on the convex side of the curve [3, 13], and 75.9%–77.2% of trunks tilted toward the side opposite the disc herniation [10, 11]. In our study, 86.9% (206/237) of disc herniations occurred on the convex side, and 86.5% (205/237) of trunks tilted toward the side opposite the disc herniation, consistent with previous findings. Zhu et al. [13] proposed an “avoidance” theory, whereby the trunk tilts toward the side opposite to the disc herniation to redistribute leg loading; reduced weight‐bearing on the affected leg can lessen nerve root irritation. We agree with this theory and further refine this mechanism: in addition to gravitational factors, the convex side of the lumbosacral scoliosis could enlarge the intervertebral foramen, which might relieve nerve compression.

For physicians, it is rather more important to select the appropriate treatment for sciatic scoliosis secondary to LDH. In this regard, we hold the same opinion as previous studies [10, 13, 18, 20]: early decompression can provide a greater opportunity for the spontaneous correction of scoliosis. Tu et al. [14] treated 20 patients under age 20 with sciatic scoliosis by IELD and found no significant differences in operation time, hospital stay, complications, or recurrences between patients with and without scoliosis. Yang et al. [18] compared the outcomes of 53 patients with single‐segment LDH accompanied by scoliosis and 21 patients with double‐segment LDH accompanied by scoliosis who underwent PELD; they found 92.5% of single‐segment and 90.5% of double‐segment patients achieved normal within 12 months. However, there was no comparative study on the treatment of LDH with sciatic scoliosis between IELD and TELD.

Our study first compared the clinical and radiographic outcomes of different approaches of PELD for the treatment of LDH with sciatic scoliosis. In this study, we observed significant improvements in both coronal and sagittal alignment after surgery. Both groups demonstrated a remarkable reduction in CBD, with near‐complete normalization at the final follow‐up. The CBD, which was initially 29.2 ± 19.0 mm in the IELD group and 29.6 ± 19.5 mm in the TELD group, decreased significantly to 1.2 ± 0.9 mm and 1.2 ± 0.8 mm, respectively. This finding aligns with previous studies [17, 21] showing that PELD can effectively address the coronal deformity associated with sciatic scoliosis. Similarly, sagittal alignment was also restored, with both groups achieving near‐normal values for sagittal balance. The SVA, a key indicator of sagittal imbalance [22], improved significantly from preoperative values of 61.5 ± 54.7 mm (IELD) and 69.5 ± 61.8 mm (TELD) to 3.4 ± 3.1 mm and 3.7 ± 3.5 mm, respectively, with all patients achieving sagittal plane balance by the final follow‐up. Additionally, TK and LL were significantly improved, indicating that PELD not only corrects the deformity but also promotes the restoration of the spine's natural curvature. The improvement in TK and LL suggests that PELD has a broader effect on spinal alignment, beyond merely addressing the specific deformities of sciatic scoliosis. These findings are consistent with those of Wang et al. [11] and Wu et al. [19]. At the same time, we have noticed that there were no significant differences between the IELD and TELD groups in both coronal and sagittal parameters at each follow‐up. This indicates that the different approaches do not affect the radiographic evolution of sciatic scoliosis.

In addition, we found that the ODI, VAS for back pain, and leg pain of each group were significantly improved after surgery and remained stable during the follow‐up period, which is consistent with the previous studies. Notably, IELD was associated with a shorter operation time and fewer intraoperative fluoroscopic examinations than TELD. This may be due to the relatively narrow intervertebral foramina in the lower lumbar spine, which may require further enlargement of the foramina [23]. At the same time, because operation time was measured from skin incision to wound closure, the time required for fluoroscopic localization was included in the operative duration. The greater number of fluoroscopic examinations required during TELD may therefore have contributed to the longer operative time. Nevertheless, the present study did not record cumulative fluoroscopy time or the duration of individual localization steps, and the difference in operative duration should not be attributed solely to fluoroscopy. TELD may require repeated trajectory adjustment, confirmation of needle and working‐cannula position, and, in selected cases, foraminoplasty. These technical steps may become more demanding when coronal deformity persists in the prone position and alters the usual fluoroscopic anatomical orientation. Consequently, the difference in operative duration is likely multifactorial. However, no significant differences were observed in intraoperative blood loss and postoperative length of stay.

Moreover, the technical learning curves of the two procedures should also be considered. Approach selection should be pathology‐directed. TELD provides a lateral and relatively ventral route to the disc and may be particularly suitable for foraminal, extraforaminal, or extreme lateral disc prolapse, in which direct access to the lateral pathology is desirable. Local anesthesia can also be used in selected patients, allowing intraoperative communication. TELD requires accurate three‐dimensional trajectory planning and may involve repeated fluoroscopic confirmation during needle placement, dilation, working‐cannula positioning, and foraminoplasty. In patients with marked sciatic scoliosis, residual deformity in the prone position may distort the usual anatomical orientation and increase the difficulty of targeting the intervertebral foramen. IELD may provide a more direct surgical corridor, particularly at L5/S1, but requires familiarity with interlaminar anatomy and careful manipulation around the traversing nerve root and dural sac. This posterior route is less affected by iliac crest height, and the relatively wide L5/S1 interlaminar window may facilitate treatment of central or paramedian herniation and selected migrated fragments. IELD also requires careful management of the ligamentum flavum and is not necessarily the preferred corridor for a purely foraminal or extraforaminal herniation. Segmental anatomy is an important consideration in selecting an endoscopic approach. TELD at L5/S1 may be technically restricted by a high iliac crest, a large L5 transverse process, a narrow neural foramen, a steep disc inclination, or unfavorable facet morphology. These anatomical constraints may require modification of the puncture trajectory or additional foraminoplasty. By contrast, the relatively wide interlaminar window at L5/S1 may provide a more direct route for IELD. Although foraminoplasty may overcome some of these obstacles, it can increase technical complexity and fluoroscopic requirements. Nevertheless, these anatomical considerations are not absolute indications or contraindications. Advances in trajectory planning and foraminoplasty techniques allow TELD to be performed in selected anatomically challenging cases. Radiation exposure is particularly relevant because the present cohort included adolescents and young adults. The lower number of fluoroscopic examinations observed in the IELD group may represent a potential advantage by reducing cumulative radiation exposure to patients and operating‐room personnel. Thus, the strengths of TELD and IELD should be regarded as complementary rather than hierarchical. In patients with LDH‐associated sciatic scoliosis, the approach should be selected according to the disc location, migration pattern, segmental anatomy, patient factors, surgeon expertise, and the learning curve of each technique.

## 5 Limitations

First, this is a retrospective study without random allocation, which may introduce selection bias. Second, the surgical approach was selected mainly according to the surgeon's experience, potentially affecting group comparability. Third, postoperative CT and flexion–extension radiographs were not routinely obtained, limiting the evaluation of bony resection, facet preservation, and lumbar stability. Future prospective studies with standardized imaging and longer follow‐up are needed.

## 6 Conclusions

Both IELD and TELD are effective, safe, and minimally invasive treatments for LDH with sciatic scoliosis. After the relief of nerve compression, the coronal and sagittal spinal alignment can be spontaneously restored to balance. IELD may have advantages in terms of shorter operation time and fewer intraoperative fluoroscopic times. However, TELD may be more appropriate for foraminal, extraforaminal, or extreme lateral pathology and can be advantageous when local anesthesia is desirable. Early identification and selection of an appropriate approach for decompression may provide greater opportunities for the spontaneous correction of scoliosis.

## Author Contributions

**Shicheng Zheng:** conceptualization, methodology, software, data curation, investigation, validation, writing – original draft, formal analysis. **Xincheng Cai:** investigation, formal analysis. **Bolu Zhang:** investigation, formal analysis. **Qinghua Zhao:** writing – review and editing, validation, supervision, project administration. **Zongshan Hu:** validation, project administration, writing – review and editing. **Bin Wang:** funding acquisition, visualization, project administration, resources. **Zezhang Zhu:** funding acquisition, visualization, project administration, resources, supervision, formal analysis. **Yong Qiu:** formal analysis, resources, supervision, project administration, visualization, funding acquisition. **Xu Sun:** funding acquisition, visualization, project administration, resources, supervision, formal analysis, validation, writing – review and editing.

## Funding

This work was supported by the Clinical Trials from Nanjing Drum Tower Hospital, Affiliated Hospital of Medical School, Nanjing University (2024‐LCYJ‐MS‐04), Clinical Trials from Nanjing Drum Tower Hospital, Affiliated Hospital of Medical School, the Nanjing University (2024‐638), the New Clinical Technologies from the Affiliated Drum Tower Hospital, Medical School of Nanjing University (XJSFZLX202303), and Jiangsu Provincial Medical Innovation Center of Orthopedic Surgery (CXZX202214).

## Disclosure

S.Z., X.C., B.Z., Q.Z., Z.H., B.W., Z.Z., Y.Q., and X.S. declare that all authors listed meet the authorship criteria according to the latest guidelines of the International Committee of Medical Journal Editors and that all authors are in agreement with the manuscript.

## Consent

Informed consent was obtained from all participants prior to their inclusion in the study.

## Conflicts of Interest

The authors declare no conflicts of interest.

## Supporting information


**Table S1:** Comparison of perioperative data in L4/5 subgroup.
**Table S2:** Comparison of radiographic evolution in L4/5 subgroup.
**Table S3:** Comparison of patient‐reported outcomes in L4/5 subgroup.
**Table S4:** Comparison of perioperative data in L5/S1 subgroup.
**Table S5:** Comparison of radiographic evolution in L4/5 subgroup.
**Table S6:** Comparison of patient‐reported outcomes in L5/S1 subgroup.

## Data Availability

The data that support the findings of this study are available on request from the corresponding author. The data are not publicly available due to privacy or ethical restrictions.
