# Supplementary material for: Comparison Between Interlaminar and Transforaminal Endoscopic Lumbar Discectomy for Sciatic Scoliosis Secondary to Lumbar Disc Herniation: A Retrospective Study With a Minimum 1 Year Follow‐Up
Source: Orthop Surg. 2026 Oct 16:10.1111/os.70447. Online ahead of print. doi: 10.1111/os.70447 (PMC13674043; doi:10.1111/os.70447)
Supplement: Supplementary file 1 [file OS-9999-0-s001.docx]

**Supplementary Table 1** Comparison of perioperative data in L4/5 subgroup

| **Variables** | **IELD group (N=101)** | **TELD group (N=83)** | ***t* value** | ***P* value** |
| --- | --- | --- | --- | --- |
| **Operation time (min)** | 88.1 ± 14.4 | 95.6 ± 23.3 | -2.34 | 0.021* |
| **Intraoperative fluoroscopy times (n)** | 4.9 ± 0.7 | 10.3 ± 2.7 | -18.33 | <0.001* |
| **Intraoperative blood loss (ml)** | 14.0 ± 7.1 | 11.6 ± 7.1 | 2.04 | 0.058 |
| **Postoperative length of stay (days)** | 2.4 ± 0.6 | 2.5 ± 0.7 | -0.92 | 0.358 |

**Notes:** **P*<0.05.

**Supplementary Table 2** Comparison of radiographic evolution in L4/5 subgroup

| **Variables** | **IELD group (N=101)** | **TELD group (N=83)** | ***t* value** | ***P* value** |
| --- | --- | --- | --- | --- |
| **PI (°)** | 49.0 ± 9.6 | 49.6 ± 9.8 | -0.31 | 0.757 |
| **Cobb angle (°)** |  |  |  |  |
| Preoperative | 14.6 ± 3.5 | 14.7 ± 3.4 | -0.14 | 0.892 |
| 3-month follow-up | 4.9 ± 3.2 | 4.6 ± 2.5 | 0.57 | 0.568 |
| 12-month follow-up | 1.4 ± 1.1 | 1.3 ± 1.0 | 0.53 | 0.600 |
| Final follow-up | 1.2 ± 0.9 | 1.2 ± 0.8 | 0.03 | 0.979 |
| **AVT (mm)** |  |  |  |  |
| Preoperative | 36.7 ± 13.7 | 38.6 ± 26.7 | -0.45 | 0.656 |
| 3-month follow-up | 12.6 ± 7.7 | 12.3 ± 7.2 | 0.24 | 0.809 |
| 12-month follow-up | 4.2 ± 3.4 | 4.6 ± 4.1 | -0.52 | 0.604 |
| Final follow-up | 2.7 ± 1.1 | 2.8 ± 0.9 | -0.22 | 0.826 |
| **CBD (mm)** |  |  |  |  |
| Preoperative | 26.5 ± 15.9 | 29.7 ± 19.6 | -0.94 | 0.347 |
| 3-month follow-up | 9.3 ± 6.7 | 9.3 ± 7.4 | -0.05 | 0.960 |
| 12-month follow-up | 2.0 ± 0.4 | 2.6 ± 0.6 | -0.96 | 0.339 |
| Final follow-up | 0.9 ± 0.3 | 1.2 ± 0.8 | -0.83 | 0.408 |
| **SVA (mm)** |  |  |  |  |
| Preoperative | 68.3 ± 26.9 | 71.0 ± 22.3 | -1.22 | 0.225 |
| 3-month follow-up | 25.5 ± 18.7 | 27.6 ± 10.6 | -0.44 | 0.664 |
| 12-month follow-up | 5.1 ± 1.3 | 6.3 ± 1.7 | -0.97 | 0.333 |
| Final follow-up | 3.0 ± 0.5 | 3.5 ± 0.7 | -0.84 | 0.403 |
| **TK (°)** |  |  |  |  |
| Preoperative | 19.5 ± 11.2 | 21.1 ± 11.5 | -0.82 | 0.416 |
| 3-month follow-up | 24.9 ± 8.4 | 25.8 ± 9.0 | -0.55 | 0.581 |
| 12-month follow-up | 30.7 ± 5.3 | 31.3 ± 5.7 | -0.60 | 0.547 |
| Final follow-up | 32.1 ± 4.5 | 32.3 ± 5.0 | -0.25 | 0.806 |
| **LL (°)** |  |  |  |  |
| Preoperative | 25.1 ± 15.5 | 23.8 ± 13.8 | 0.48 | 0.629 |
| 3-month follow-up | 33.1 ± 8.0 | 32.4 ± 9.0 | 0.45 | 0.651 |
| 12-month follow-up | 42.5 ± 4.5 | 42.4 ± 4.6 | 0.12 | 0.907 |
| Final follow-up | 42.7 ± 4.3 | 42.8 ± 4.2 | -0.15 | 0.884 |

**Supplementary Table 3** Comparison of patient-reported outcomes in L4/5 subgroup

| **Variables** | **IELD group (N=101)** | **TELD group (N=83)** | ***t* or *χ²* value** | ***P* value** |
| --- | --- | --- | --- | --- |
| **VAS for back pain** |  |  |  |  |
| Preoperative | 6.4 ± 0.6 | 6.6 ± 0.9 | -1.75 | 0.082 |
| 3-month follow-up | 2.5 ± 1.6 | 2.3 ± 1.6 | 0.85 | 0.397 |
| 6-month follow-up | 1.5 ± 1.1 | 1.4 ± 1.2 | 0.21 | 0.835 |
| 12-month follow-up | 1.2 ± 0.3 | 1.1 ± 1.4 | 0.31 | 0.757 |
| Final follow-up | 1.0 ± 0.1 | 0.9 ± 0.6 | 0.64 | 0.526 |
| **VAS for leg pain** |  |  |  |  |
| Preoperative | 7.1 ± 1.9 | 7.3 ± 1.8 | -0.73 | 0.468 |
| 3-month follow-up | 2.0 ± 1.3 | 2.0 ± 1.5 | -0.04 | 0.968 |
| 6-month follow-up | 1.8 ± 1.0 | 1.6 ± 0.9 | 1.22 | 0.226 |
| 12-month follow-up | 1.3 ± 0.4 | 1.2 ± 0.4 | 0.42 | 0.674 |
| Final follow-up | 1.2 ± 0.3 | 1.1 ± 0.4 | 0.33 | 0.739 |
| **ODI (%)** |  |  |  |  |
| Preoperative | 52.6 ± 4.6 | 52.6 ± 5.1 | -0.09 | 0.928 |
| 3-month follow-up | 15.8 ± 2.9 | 16.0 ± 3.0 | -0.41 | 0.682 |
| 6-month follow-up | 9.7 ± 2.8 | 9.3 ± 2.7 | 0.87 | 0.384 |
| 12-month follow-up | 7.9 ± 1.3 | 8.0 ± 1.2 | -0.58 | 0.562 |
| Final follow-up | 6.7 ± 1.4 | 6.8 ± 1.4 | -0.44 | 0.664 |
| **Modified MacNab evaluation, n (%)** |  |  | 0.15 | 0.930 |
| Excellent | 24 (53.3) | 54 (54.6) |  |  |
| Good | 18 (40.0) | 37 (37.4) |  |  |
| Fair | 3 (6.7) | 8 (8.1) |  |  |
| Poor | 0 (0.0) | 0 (0.0) |  |  |
| Excellent/good rate (%) | 93.3 | 92.0 |  |  |

**Supplementary Table 4** Comparison of perioperative data in L5/S1 subgroup

| **Variables** | **IELD group (N=33)** | **TELD group (N=20)** | ***t* value** | ***P* value** |
| --- | --- | --- | --- | --- |
| **Operation time (min)** | 86.7 ± 18.8 | 110.0 ± 21.2 | -2.41 | 0.018 * |
| **Intraoperative fluoroscopy times (n)** | 5.0 ± 0.7 | 10.5 ± 1.7 | -6.35 | 0.008 * |
| **Intraoperative blood loss (ml)** | 12.6 ± 8.0 | 10.0 ± 7.1 | 0.64 | 0.523 |
| **Postoperative length of stay (days)** | 2.5 ± 0.6 | 3.8 ± 1.2 | -1.13 | 0.340 |

**Notes:** **P*<0.05.

**Supplementary Table 5** Comparison of radiographic evolution in L4/5 subgroup

| **Variables** | **IELD group (N=33)** | **TELD group (N=20)** | ***t* value** | ***P* value** |
| --- | --- | --- | --- | --- |
| **PI (°)** | 47.3 ± 7.8 | 48.0 ± 7.1 | -0.17 | 0.866 |
| **Cobb angle (°)** |  |  |  |  |
| Preoperative | 14.9 ± 3.6 | 13.5 ± 0.6 | 1.60 | 0.113 |
| 3-month follow-up | 3.8 ± 1.7 | 2.5 ± 1.7 | 1.58 | 0.118 |
| 12-month follow-up | 1.1 ± 0.9 | 1.0 ± 0.4 | 0.69 | 0.495 |
| Final follow-up | 0.9 ± 0.7 | 0.8 ± 0.3 | -1.45 | 0.150 |
| **AVT (mm)** |  |  |  |  |
| Preoperative | 36.7 ± 24.3 | 28.9 ± 16.3 | 0.63 | 0.532 |
| 3-month follow-up | 10.2 ± 6.5 | 8.2 ± 3.2 | 0.60 | 0.548 |
| 12-month follow-up | 3.7 ± 2.8 | 2.8 ± 1.9 | 0.62 | 0.538 |
| Final follow-up | 2.7 ± 1.9 | 2.1 ± 1.4 | 0.64 | 0.523 |
| **CBD (mm)** |  |  |  |  |
| Preoperative | 30.5 ± 20.4 | 28.4 ± 18.9 | 0.21 | 0.834 |
| 3-month follow-up | 8.2 ± 2.8 | 7.1 ± 2.5 | 0.31 | 0.755 |
| 12-month follow-up | 2.3 ± 0.3 | 1.5 ± 0.8 | 0.65 | 0.516 |
| Final follow-up | 0.9 ± 0.2 | 1.0 ± 0.2 | -0.18 | 0.855 |
| **SVA (mm)** |  |  |  |  |
| Preoperative | 63.1 ± 28.4 | 62.6 ± 28.6 | 1.03 | 0.307 |
| 3-month follow-up | 24.6 ± 17.5 | 26.2 ± 12.0 | 1.61 | 0.112 |
| 12-month follow-up | 5.3 ± 2.7 | 3.0 ± 1.5 | 0.68 | 0.497 |
| Final follow-up | 3.2 ± 0.8 | 4.4 ± 0.7 | -0.65 | 0.517 |
| **TK (°)** |  |  |  |  |
| Preoperative | 21.6 ± 12.1 | 31.0 ± 6.1 | -1.54 | 0.128 |
| 3-month follow-up | 26.4 ± 8.1 | 32.8 ± 4.6 | -1.56 | 0.122 |
| 12-month follow-up | 31.1 ± 4.9 | 31.5 ± 3.4 | -0.17 | 0.866 |
| Final follow-up | 31.9 ± 4.5 | 31.5 ± 3.4 | 0.19 | 0.847 |
| **LL (°)** |  |  |  |  |
| Preoperative | 24.1 ± 14.4 | 36.5 ± 11.7 | -1.70 | 0.093 |
| 3-month follow-up | 33.4 ± 8.7 | 41.0 ± 6.8 | -1.72 | 0.090 |
| 12-month follow-up | 41.7 ± 4.9 | 45.0 ± 3.5 | -1.32 | 0.189 |
| Final follow-up | 41.7 ± 4.9 | 45.0 ± 3.6 | -1.32 | 0.191 |

**Supplementary Table 6** Comparison of patient-reported outcomes in L5/S1 subgroup

| **Variables** | **IELD group (N=33)** | **TELD group (N=20)** | ***t* or *χ²* value** | ***P* value** |
| --- | --- | --- | --- | --- |
| **VAS for back pain** |  |  |  |  |
| Preoperative | 6.5 ± 0.8 | 6.3 ± 0.5 | 0.50 | 0.621 |
| 3-month follow-up | 2.4 ± 1.3 | 1.5 ± 0.5 | 1.29 | 0.199 |
| 6-month follow-up | 1.3 ± 1.0 | 1.0 ± 0.8 | 0.60 | 0.549 |
| 12-month follow-up | 1.2 ± 0.7 | 1.0 ± 0.8 | 0.02 | 0.986 |
| Final follow-up | 1.1 ± 0.3 | 1.0 ± 0.2 | 0.12 | 0.906 |
| **VAS for leg pain** |  |  |  |  |
| Preoperative | 7.1 ± 2.0 | 6.7 ± 3.2 | 0.33 | 0.738 |
| 3-month follow-up | 1.8 ± 1.0 | 1.5 ± 0.6 | 0.65 | 0.515 |
| 6-month follow-up | 1.6 ± 0.9 | 1.3 ± 0.5 | 0.73 | 0.465 |
| 12-month follow-up | 1.12± 0.3 | 0.8 ± 0.9 | 0.63 | 0.530 |
| Final follow-up | 1.0 ± 0.1 | 0.7 ± 0.3 | 0.55 | 0.586 |
| **ODI (%)** |  |  |  |  |
| Preoperative | 52.2 ± 4.8 | 51.7 ± 4.1 | 0.20 | 0.845 |
| 3-month follow-up | 15.9 ± 3.1 | 14.2 ± 0.9 | 1.10 | 0.276 |
| 6-month follow-up | 9.3 ± 3.1 | 9.5 ± 0.5 | -0.24 | 0.812 |
| 12-month follow-up | 7.9 ± 1.0 | 8.5 ± 1.2 | -0.94 | 0.352 |
| Final follow-up | 7.0 ± 1.3 | 6.2 ± 1.5 | 1.19 | 0.238 |
| **Modified MacNab evaluation, n (%)** |  |  | 1.84 | 0.398 |
| Excellent | 39 (43.8) | 3 (75.0) |  |  |
| Good | 43 (48.3) | 1 (25.0) |  |  |
| Fair | 7 (7.9) | 0 (0.0) |  |  |
| Poor | 0 (0.0) | 0 (0.0) |  |  |
| Excellent/good rate (%) | 91.1 | 90 |  |  |
